# *Achyrocline satureioides* (Lam.) DC (Asteraceae) Extract-Loaded Nanoemulsions as a Promising Topical Wound Healing Delivery System: In Vitro Assessments in Human Keratinocytes (HaCaT) and HET-CAM Irritant Potential

**DOI:** 10.3390/pharmaceutics13081241

**Published:** 2021-08-12

**Authors:** Lucélia Albarello Balestrin, Tainá Kreutz, Flávia Nathiely Silveira Fachel, Juliana Bidone, Nicolly Espindola Gelsleichter, Letícia Scherer Koester, Valquiria Linck Bassani, Elizandra Braganhol, Cristiana Lima Dora, Helder Ferreira Teixeira

**Affiliations:** 1 Programa de Pós-Graduação em Ciências Farmacêuticas, Universidade Federal do Rio Grande do Sul, Porto Alegre 90610-000, Brazil; luceliaalbarello@gmail.com (L.A.B.); tainakreutz@gmail.com (T.K.); flavia.fachel@ufrgs.br (F.N.S.F.); leticia.koester@ufrgs.br (L.S.K.); valquiria.bassani@ufrgs.br (V.L.B.); 2 Curso de Farmácia, Centro de Ciências Químicas, Farmacêuticas e de Alimentos, Universidade Federal de Pelotas, Pelotas 96010-900, Brazil; julianabidone@gmail.com; 3 Programa de Pós-Graduação em Biociências, Universidade Federal de Ciências da Saúde de Porto Alegre, Porto Alegre 90050-170, Brazil; nicolly_3@hotmail.com (N.E.G.); elizbraganhol@yahoo.com.br (E.B.); 4 Instituto de Cardiologia do Rio Grande do Sul/Fundação Universitária do Instituto de Cardiologia (IC-FUC), Porto Alegre 90040-371, Brazil; 5 Programa de Pós-Graduação em Ciências da Saúde, Laboratório de Nanotecnologia, Faculdade de Medicina, Universidade Federal do Rio Grande, Rio Grande 96203-900, Brazil; cristianadora@gmail.com

**Keywords:** *Achyrocline satureioides* extract, flavonoids, nanoemulsion, topical nanosystem, wound healing, cell viability and migration, human keratinocytes (HaCaT), HET-CAM

## Abstract

*Achyrocline satureioides* (Lam.) DC Asteraceae extracts (ASEs) have been investigated for the treatment of various skin disorders. This study reports the effects of ASE-loaded nanoemulsions (NE_ASE_) on the cellular viability, death by necrosis, and migration of immortalized human keratinocytes (HaCaT cell line), as well as the irritant potential through the hen’s egg chorioallantoic membrane test (HET-CAM). NE_ASE_ exhibited a polydispersity index above 0.12, with a droplet size of 300 nm, ζ-potential of −40 mV, and content of flavonoids close to 1 mg/mL. No cytotoxicity of the ASE was observed on HaCaT by MTT assay (up to 10 µg/mL). A significant increase of HaCaT viability was observed to NE_ASE_ (up to 5 μg/mL of flavonoids), compared to treatment with the ASE. The necrosis death evaluation demonstrated that only NE_ASE_ did not lead to cell death at all the tested concentrations. The scratch assay demonstrated that NE_ASE_ was able to increase the cell migration at low flavonoid concentrations. Finally, the HET-CAM test proved the non-irritative potential of NE_ASE_. Overall, the results indicate the potential of the proposed formulations for topical use in wound healing, in view of their promising effects on proliferation and migration in keratinocytes, combined with an indication of the absence of cytotoxicity and non-irritating potential.

## 1. Introduction

A considerable number of medicines that are based on bioactive compounds from medicinal plants are available on the pharmaceutical market. Such medicines contain drugs isolated from medicinal plant extracts or synthesized from compounds identified from these plant extracts [1]. However, despite the difficulties in relating the pharmacological activity with a specific compound or class of compounds and in elucidating the mechanism of action involved, phytomedicines containing crude plant extracts are widely used in clinical practice [2]. These products contain a variety of compounds that can interact with multiple targets, leading to additive and/or synergistic effects. Such effects have been widely suggested in well-documented and widely discussed scientific literature [3,4,5,6].

Extracts of *Achyrocline satureioides* (Lam.) DC (Asteraceae) (ASEs), a native medicinal plant in the southeast region of South America, have been widely investigated in different in vitro and in vivo studies [7]. To date, the potential activity of these extracts for various skin disorders has been widely described in view of the promising results obtained in different biological models, including antioxidant [8], anti-inflammatory [9,10], and antiherpes properties [11,12]. In a preliminary study with plants popularly used for wound healing, both aqueous by decoction and ethanolic by maceration (3 × 24 h) ASEs showed the ability to increase cell proliferation in keratinocyte (HaCaT) and fibroblast (MRC-5) cell lines [13]. These results are promising as a stimulus in cell proliferation in vitro is indicative of a possible healing activity for this extract, which corroborates the arguments for its traditional use. Pereira and co-workers (2017) [14] demonstrated in vivo that ASE, when incorporated into an ointment, was able to promote a better renewal of collagen compared to the control groups, which is essential for complete wound healing. 

The presence of several phytochemicals has been described in ASE extracts, including phenolic acids, chalcones, coumarins, polysaccharides, and polyacetylenes. Nevertheless, in most cases, the biological activities have been related to the presence of the flavonoid aglycones quercetin (QCT), luteolin (LUT), and 3-*O*-methylquercetin (3MQ) in the extracts obtained from the aerial parts of the plant, using organic solvents [8,10,15,16,17]. However, the poor water solubility of the flavonoid aglycones could limit their incorporation into topical products (generally presenting a hydrophilic nature), as well as the permeation and retention of these compounds through the skin. To circumvent this drawback, our research group has patented an original procedure to incorporate the main flavonoid aglycones from ASE into lipid-nanoemulsions by means of a spontaneous emulsification procedure [18]. This procedure allows the simultaneous incorporation of QCT, LUT, and 3MQ into monodispersed nanosized emulsions (200 nm range) [19]. Previous results have shown the distribution and accumulation of these flavonoids in porcine ear skin layers from nanoemulsions using Franz-type diffusion cells [12,20].

In this study, we present new evidence of the potentiation of the healing activity of this extract by its incorporation into a topical nanosystem. We investigated in vitro the effect of an ASE-loaded nanoemulsion on cell viability by MTT assay, cell death by necrosis by means of propidium iodate incorporation, and cell migration by the scratch assay in keratinocytes (HaCaT cell line). Finally, the irritant potential was determined with the hen’s egg chorioallantoic membrane test (HET-CAM).

## 2. Materials and Methods

### 2.1. Materials and Reagents

Egg-lecithin (Lipoid E-80^®^), polysorbate 80, and vitamin E were obtained from Lipoid GmbH (Ludwigshafen, Germany). Dulbecco’s modified Eagle’s medium (DMEM), 3,(4,5-dimethyl)-2,5diphenyl-tetrazolium bromide salt (MTT), and propidium iodide (PI) were purchased from Sigma-Aldrich Co. (St. Louis, MO, USA). Fetal bovine serum (FBS) and trypsin solution were obtained from Gibco (Grand Island, NE, USA). Methanol (J.T. Baker, Center Valley, PA, USA), acetonitrile (Tedia Brasil, Rio de Janeiro, Brazil), and phosphoric acid (Merck, Darmstadt, Germany) were of analytical grade and were used for the analysis by HPLC.

Dried inflorescences of *Achyrocline satureioides* were acquired from Centro Pluridisciplinar de Pesquisas Químicas, Biológicas e Agrícolas (CPQBA) da Universidade Estadual de Campinas (São Paulo, Brazil), and a sample of the species was deposited in the herbarium of the same institution (number 308). The research with genetic material from Brazilian biodiversity was registered in the SisGen (Sistema Nacional de Gestão do Patrimônio Genético e do Conhecimento Tradicional Associado) with number A8CA9A8.

### 2.2. Preparation of A. satureioides Extract (ASE)

Initially, the inflorescences of the plant were manually cleaned and ground in a hammer mill. The ASE was obtained by maceration in ethanol 80% (*v*/*v*) for a period of eight days using a plant:solvent ratio of 7.5% (*w*/*v*), according to the method of Bidone et al. (2014) [19]. The extract was then pressed, filtered, and stored in the dark at −20 °C. The final content of the extract was 1 mg/mL of total flavonoids.

### 2.3. Flavonoid Determination by Ultra-Fast Liquid Chromatography (UFLC)

The content of flavonoids QCT, LUT, and 3MQ was determined in the ASE and the developed formulation (as total flavonoids) using validated ultra-fast liquid chromatography (UFLC), as previously described by Balestrin et al. (2020) [21]. The analyses were performed on a Shimadzu Prominence system device coupled with photodiode array (PDA) detection and an automatic injector controlled by LC-Solution Multi PDA software (Kyoto, Japan). The stationary phase was composed of a Phenomenex Luna column C18 (Phenomenex, 100 × 2.0 mm i.d.; particle size 2.5 µm) guarded by an in-line pre-column Ultra KrudKatcher filter (Phenomenex, Torrance, CA, USA). The mobile phase consisted of methanol:phosphoric acid 1% (48:52) in isocratic mode. The flow of the mobile phase was 0.3 mL/min, and the volume of injection was 4 µL. The wavelength was adjusted to 362 nm, and the analysis was carried out at 40 °C.

### 2.4. Preparation of A. satureioides Extract-Loaded Nanoemulsions

Nanoemulsions containing ASE (NE_ASE_) were prepared by a spontaneous emulsification method, as described by Bidone et al. (2014) [19]. Briefly, the organic phase, consisting of *A. satureioides* ethanolic extract, egg-lecithin, vitamin E, fixed oil (medium chain triglycerides), and ethanol, was poured into the aqueous phase, composed of polysorbate 80 and water, under magnetic stirring for 30 min. Afterwards, the ethanol was removed, and the formulation was concentrated by evaporation under reduced pressure at 40 °C, up to 1% (*w*/*v*) of *A. satureioides* dry residue. Nanoemulsions without extract (blank nanoemulsion—NE_B_) were also prepared as a control.

### 2.5. Physicochemical Characterization of the Nanoemulsions

NE_ASE_ and NE_B_ formulations were characterized according to their physical appearance, mean droplet size, polydispersity index, ζ-potential, and viscosity and transmission electron microscopy (TEM). The mean droplet size and polydispersity index were determined by photon correlation spectroscopy at 25 °C, and the ζ-potential was determined by electrophoretic mobility at 25 °C. The analyses were performed using Zetasizer Nano-ZS90^®^ equipment (Malvern Instruments, Malvern, UK) after dilution of the samples in water and 1 mM NaCl solution, respectively. The viscosity was performed by capillary viscometry using an Ostwald viscometer (20 °C ± 0.1 °C), and the morphological analysis of the formulations was conducted using transmission electron microscopy. The images were obtained using a JEM 1220EXII microscope (Jeol Ltd., Akishim, Japan).

The flavonoid content in each formulation was determined using the UFLC conditions described above (Section 2.2). Nanoemulsions were solubilized in methanol and diluted properly prior to UFLC analysis.

### 2.6. Keratinocyte Cell Culture and Treatment

HaCaT cells (immortalized human keratinocytes) were obtained from Thermo Fisher Scientific (Waltham, MA, USA). DMEM medium supplemented with 10% FBS was used for the culturing of the cells, which were maintained at 37 °C in a humidified 5% CO_2_ atmosphere. Cells were seeded in 96 and 48-well plates (8 × 10^3^ and 1.5 × 10^4^ cells per well, respectively) for the MTT, PI incorporation, and cell migration assays. The cultures were kept at 37 °C in a humidified 5% CO_2_ atmosphere for 24 h and were further treated with ASE (previously dried and resuspended in dimethyl sulfoxide (DMSO)) and NE_ASE_ at 0.625, 1.25, 2.5, 5, and 10 μg/mL concentrations. After 24 h of treatment, cell viability, death by necrosis, and migration were determined as follows.

#### 2.6.1. Cell Viability (MTT) Assay

Cell viability was assessed using the dehydrogenase-dependent MTT reduction assay. This is a colorimetric assay to determine cell metabolic activity and viability. Following the treatment, the medium was removed and MTT solution (5 mg/mL) was added to the culture media at a final concentration of 0.5 mg/mL. Cells were incubated for 60 min at 37 °C in a humidified 5% CO_2_ atmosphere. The medium was then removed, and plates were shaken with DMSO for 30 min. The absorbance was measured at 492 nm using a spectrophotometer SpectraMax^®^ M2 (Molecular Devices, San Jose, CA, USA) [22]. Controls containing DMSO (0.01%) and NE_B_ were performed. Results were expressed as the percentage of the control.

#### 2.6.2. Propidium Iodide (PI) Assay

Cell death by necrosis was analyzed with phase contrast and fluorescence microphotographs, observing morphological alterations (cell elongation and crenation) and/or PI incorporation. For this assay, cells were incubated with PI (7.5 μM) for 60 min at the end of the treatment. An inverted microscope (Olympus IX71, Olympus Corporation, Shinjuku, Tokyo, Japan) fitted with a standard rhodamine filter was excited at 515 and 560 nm using PI fluorescence. Images were captured using a digital camera connected to the microscope. Controls containing DMSO (0.01%) and NE_B_ were performed. Results were expressed as a qualitative evaluation of morphological alterations and PI incorporation.

#### 2.6.3. Cell Migration

Cell migration was investigated by the scratch-wound assay. The scratch was performed when the cell density reached 80%, using a P200 pipette tip in the cell monolayer. The cells were then treated with ASE and NE_ASE_ at 0.625, 1.25, 2.5, 5, and 10 μg/mL concentrations prepared in DMEM/0.5% FBS. A control containing DMEM/0.5% FBS was used. The scratch images were taken after 0, 6, 18, and 24 h. Images were captured using a digital camera connected to the microscope (Olympus IX71, Olympus Corporation, Shinjuku, Tokyo, Japan). ImageJ software (http://imagej.nih.gov/ij/, accessed on 2 July 2020) was employed for cell migration quantification, comparing this with the zero-time width. Four visual fields were randomly selected for each sample. The results were expressed as the mean percentage of migration in triplicates.

### 2.7. Hen’s Egg Chorioallantoic Membrane Test (HET-CAM)

Fertilized hen’s eggs were obtained from a local producer immediately after laying and were placed in an automatic rotary incubator (Chocar Chocadeiras, Conceição do Coité, Brazil) for 10 days under controlled temperature (37.8 ± 1.0 °C) and relative humidity (45–65%). On the 10th day, the eggs were candled to ensure the viability of the embryos. Defective or cracked eggs were discarded. The shell was then removed at the air cell with tweezers, and the inner membrane was withdrawn to reveal the highly vascularized chorioallantoic membrane (CAM).

Then, 0.3 mL of test substance (*n* = 5) was applied to the CAM and left in contact for 300 s. The following groups were tested: 0.9% *w*/*v* NaCl (negative control), 0.1 M NaOH (positive control for hemorrhage and coagulation), 1% *w*/*v* sodium lauryl sulfate (positive control for vasoconstriction), olive oil (negative control, applied to solubilize ASE) [23], ASE:olive oil 1:100 (*w*/*v*) (ASE solubilized in olive oil in the same proportion as found in NE_ASE_), NE_B_ (blank nanoemulsion), and NE_ASE_ (nanoemulsion containing ASE). Due to the opacity of the nanoemulsions, saline solution was used to rinse the CAM after 20 s, facilitating the observation of vascular effects. The irritation score (IS) was calculated using Equation (1):

IS = [5 × (301 − hemorrhage time in sec)/300] + [7 × (301 − vasoconstriction time in sec)/300] + [9 × (301 − coagulation time in sec)/300](1)

The IS was presented as the mean and relative standard deviation in percentage (RSD %). The statistical analysis of the IS score was performed by a one-way analysis of variance (ANOVA) followed by Tukey’s post hoc test (*p* ≤ 0.05). In addition, the damage to the hen’s egg-chorioallantoic membrane was classified by the mean as non-irritant (0–0.9), slight irritant (1.0–4.9), moderate irritant (5–8.9), and extreme irritant (9–21) [24,25,26]. 

The HET-CAM assay was approved by The Animal Use Ethics Committee from UFRGS under protocol number 40087. All procedures were performed according to the Brazilian National Animal Care Ethical Council (CONCEA) guidelines and Law No. 11794/2008 for the proper care and use of experimental animals.

### 2.8. Statistical Analysis

Results were expressed as the mean ± standard deviation of at least three independent experiments. An ANOVA was used to compare the experimental data, and the Tukey post hoc test was used to discriminate the differences at a significance level of *p* < 0.05. All analyses were conducted using Action Software (Version 2.5) or GraphPad Prism 5 Software.

## 3. Results and Discussion

### 3.1. Physicochemical Characterization of Nanoemulsions

**Figure 1:**
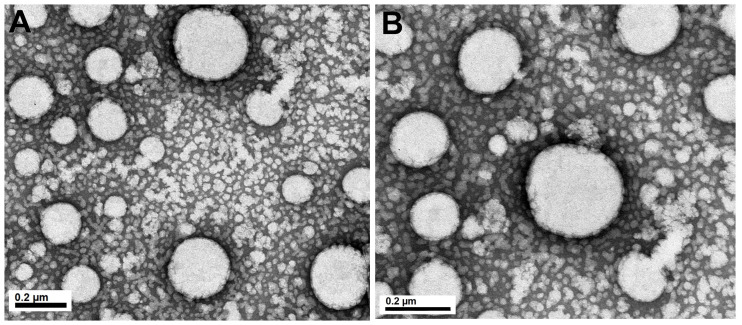
TEM images of nanoemulsion containing *A. satureioides* extract (NE_ASE_). Where: (**A**) 75 K increase and (**B**) 120 K increase.

The physicochemical properties of nanoemulsions obtained by the spontaneous emulsification procedure are presented in Table 1. This procedure yielded monodispersed nanoemulsions (PDI < 0.12) exhibiting a droplet size in a range of 200–300 nm, according to the images obtained by transmission electron microscopy displayed in Figure 1. The incorporation of ASE into nanoemulsions led to a greater average droplet diameter and viscosity of the formulations. The wide variety of the compounds from ASE associated (into the oily core and/or oil/water interface) with the colloidal structure, as well as the possible interactions of these compounds with the phospholipids from egg-lecithin, may have an effect on the physicochemical properties of the nanoemulsion [19]. A higher negative ζ-potential of NE_ASE_ (−43 mV) was noticed in comparison with NE_B_ (−21 mV). This result may be a consequence of the negatively charged phospholipids and free fatty acids in the egg yolk lecithin and the extract components located at the oil/water interface of the nanoemulsions, as phenolic acids [19].

Prior to the evaluation of the flavonoids loading with nanoemulsions, the flavonoid content (QCT, LUT, and 3MQ) was estimated in the hydroethanolic extract by the UFLC method previously described. Overall, 251.70 ± 10.86 µg/mL of QCT, 154.08 ± 6.61 µg/mL of LUT, and 687.32 ± 30.81 µg/mL of 3MQ was detected, in accordance with previous literature using similar extraction conditions [12,19]. Final formulations contained 1% (*w*/*v*) of *A. satureioides* dry residue. The flavonoid content in nanoemulsions exhibited a recovery close to 100%, indicating a satisfactory recovery for all formulations. Overall results demonstrated that, under the conditions used in this study, nanoemulsions maintained the analyzed parameters and were consistent with our previous report [19], demonstrating that the experimental conditions were well controlled.

### 3.2. In Vitro Cell Viability and Migration Evaluation in Keratinocytes 

**Figure 2:**
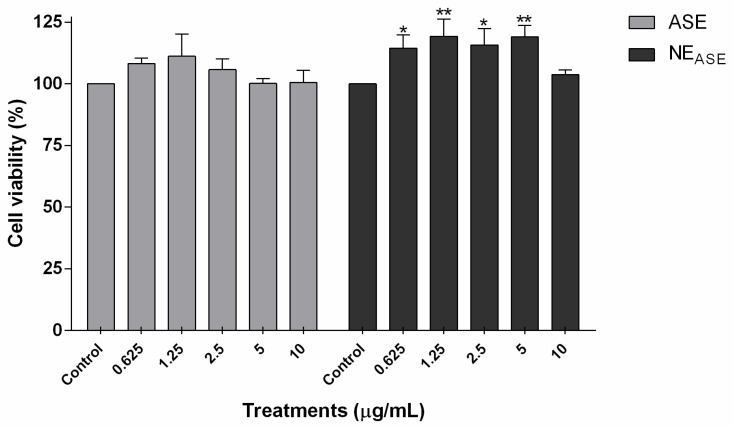
Cell viability (MTT assay) of HaCaT cells (immortalized human keratinocytes) after 24 h of treatment with ASE and NE_ASE_ at 0.625, 1.25, 2.5, 5, and 10 µg/mL concentrations. Appropriate vehicle controls with DMSO 0.01% (*v*/*v*) and NE_B_ were performed for ASE and NE_ASE_ treatments, respectively. Value represents mean ± SD for triplicate tests. Data were analyzed by a one-way analysis of variance followed by Tukey’s post hoc test. * *p* < 0.05, and ** *p* < 0.01, different from control group. ASE: *Achyrocline satureioides* extract; NE_ASE_: *Achyrocline satureioides* extract-loaded nanoemulsion; NE_B_: blank nanoemulsion.

Cell viability was determined using the MTT assay to evaluate the safety profile and the ability of ASE to induce the proliferation of human keratinocytes [27,28]. As can be seen in Figure 2, the ASE did not induce HaCaT cytotoxicity but rather a tendency to increase the cell viability of keratinocytes when compared to the control after 24 h of treatment for all concentrations tested (0.625–10 µg/mL). This result agrees with a previous report for the aqueous and ethanolic ASE, which indicated the same tendency in keratinocytes over a concentration range of ASE between 1–50 µg/mL [13]. When ASE is incorporated into a nanoemulsion (NE_ASE_), a significant increase in cell viability is noticeable up to 5 μg/mL (*p* < 0.05). These results indicate that the incorporation of ASE in nanoemulsions is able to increase the cell viability, thus increasing the proliferative capacity of these cells when compared to treatment with the free extract (ASE).

**Figure 3:**
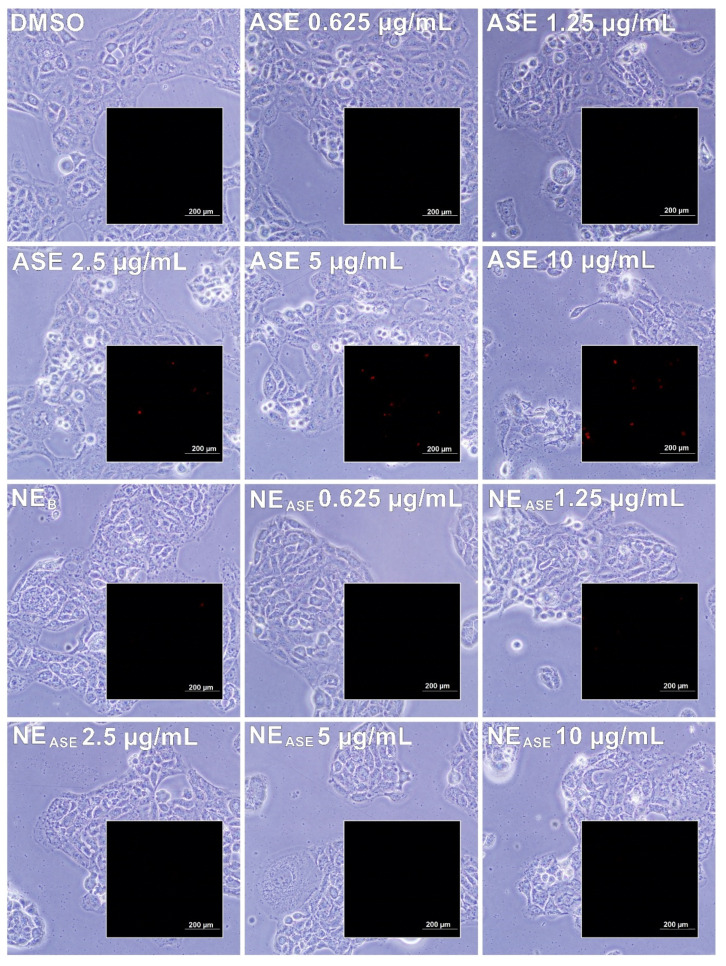
Phase contrast and fluorescence microphotographs (insert) of PI incorporation in HaCaT cells (immortalized human keratinocytes) after 24 h of treatment with ASE and NE_ASE_ at 0.625, 1.25, 2.5, 5, and 10 µg/mL concentrations. Appropriate vehicle controls with DMSO 0.01% (*v*/*v*) and NE_B_ were performed for ASE and NE_ASE_ treatments, respectively. ASE: *Achyrocline satureioides* extract; NE_ASE_: *Achyrocline satureioides* extract-loaded nanoemulsion; NE_B_: blank nanoemulsion.

Cell death by necrosis was assessed by the PI assay, which is used as a marker that permeates only damaged cell membranes. Intercalation complexes between PI and DNA are formed, which act by amplifying the fluorescence. The possibility of visualization of this fluorescence finally allows us to evaluate non-vital cells [29]. After 24 h of cell treatment with ASE and NE_ASE_ (from 0.625 to 10 µg/mL), the incorporation of PI and morphological alterations (cell elongation and crenation) for *A. satureioides*-treated cells was noticeable only at concentrations above 2.5 µg/mL (Figure 3 and Table 2), indicating that toxicity and cell death by necrosis occurred as a function of the extract concentration used. On the other hand, for the cells treated with NE_ASE_, it was not possible to observe the incorporation of PI in any of the concentrations tested, indicating that there was no damage to the cell membrane treated with the formulations. These results suggested for the first time that the incorporation of the extract into a nanoemulsion may decrease cell death by necrosis. Additionally, although cell death by apoptosis was not observed in the MTT assay, the positive effect of the incorporation of ASE in nanoemulsions on cell proliferation was also demonstrated. Accordingly, Bidone et al. (2015) [12] previously reported a decrease in the toxicity of ASE when incorporated into nanoemulsions using Vero cells.

**Figure 4:**
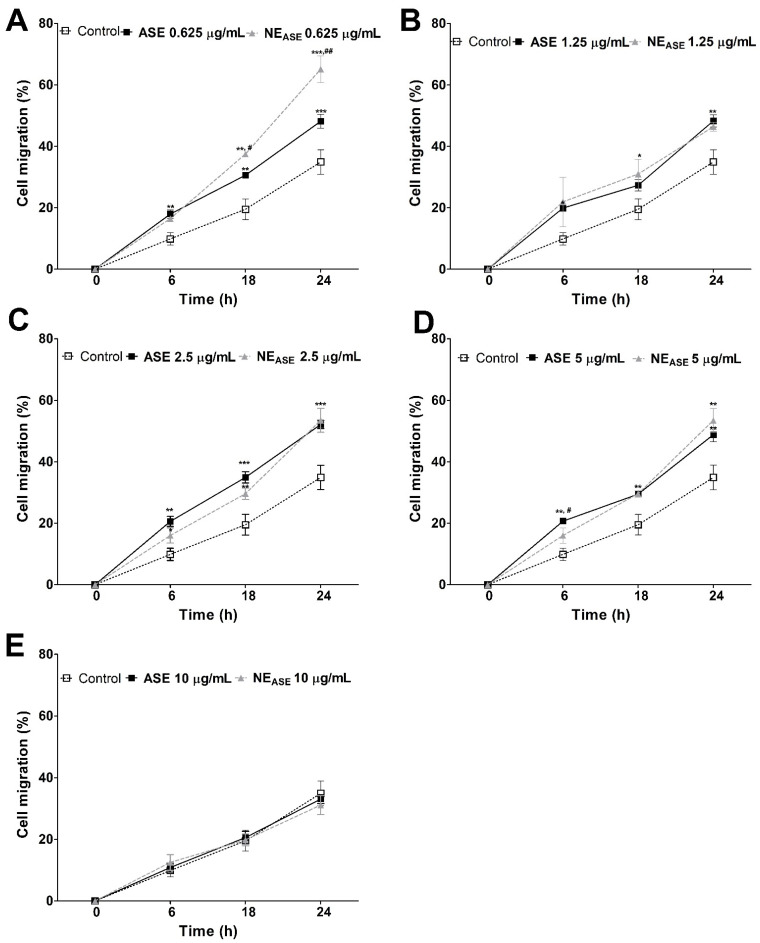
Cell migration (%) of HaCaT (immortalized human keratinocytes) after 6, 18, and 24 h of treatment with ASE and NE_ASE_ at 0.625 (**A**), 1.25 (**B**), 2.5 (**C**), 5 (**D**), and 10 (**E**) µg/mL concentrations. Untreated control group was performed. Value represent mean ± SD for triplicate tests. Data were analyzed by one-way analysis of variance followed by Tukey’s post hoc test. * *p* < 0.05, ** *p* < 0.01, and *** *p* < 0.001, different from control group; ^#^ *p* < 0.05, and ^##^ *p* < 0.01, different from ASE group. ASE: *Achyrocline satureioides* extract; NE_ASE_: *Achyrocline satureioides* extract-loaded nanoemulsion.

Cell migration has great importance in the development and physiology of diseases. The scratch-wound model is an in vitro model that is often used to study re-epithelialization because it mimics aspects of keratinocyte migration in vivo. This assay combined the effects of proliferation and migration, both of which are important in wound healing [30,31]. This study was used to evaluate the migration of human keratinocytes (HaCaT), since they are cells that are directly involved in the wound healing process [32]. After scratching, it is possible to monitor cell migration with the support of a microscope, since the cells migrate from the intact region to the scattered region; images are obtained at 0, 6, 18, and 24 h and expressed as percentage of migration. The effects of NE_ASE_ and ASE on cell migration were evaluated and can be observed in Figure 4.

At the lowest concentration used (0.625 µg/mL), NE_ASE_ exerted a positive effect on the migration of keratinocytes over the scratching when compared to ASE and the control group at 6, 18, and 24 h. From the 1.25 µg/mL concentration, there is a decrease in the migration of NE_ASE_-treated samples; this behavior is similar to ASE, but still represents a positive effect on the migration of keratinocytes in comparison to the control. However, only at the highest concentration (10 µg/mL) was there no evidence of an increase in the migration of NE_ASE_ and ASE-treated cells when compared to the control (untreated cells—DMEM/0.5% FBS). It should be noted that the scratch assay was conducted by maintaining the cells at 0.5% FBS to minimize cell proliferation. Overall, the results indicate that NE_ASE_ exerts a positive effect on cell migration, depending on the concentration, which is a key consideration, as an increased cell migration rate and wound closure may have a crucial effect on the topical wound healing activity [33].

### 3.3. Hen’s Egg Chorioallantoic Membrane Test (HET-CAM)

The HET-CAM test has been widely used as an alternative methodology to the in vivo ocular irritation test. In recent years, it has also been employed as a preliminary screening test before in vivo study to provide the irritant potential of substances on membranes and skin [24,26,34]. 

**Figure 5:**
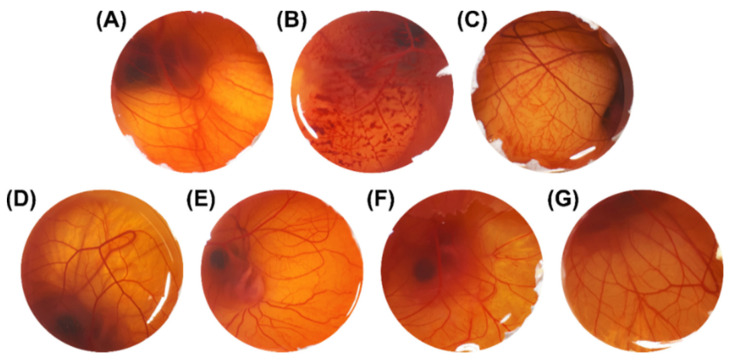
Images illustrating the effects of different substances applied on the chorioallantoic membrane over a 5 min period (1× magnification)**.** (**A**) NaCl 0.9% *w*/*v*, (**B**) NaOH 0.1 M, (**C**) Sodium lauryl sulfate 1% *w*/*v*, (**D**) Olive oil (negative control), (**E**) ASE solubilized in olive oil, (**F**) NE_B_, (**G**) NE_ASE_. ASE: *Achyrocline satureioides* extract; NE_B_: blank nanoemulsion; NE_ASE_: *Achyrocline satureioides* extract-loaded nanoemulsion.

Results for irritant potential are shown in Figure 5 and Table 3. Before testing the ASE and the developed nanoemulsions, positive and negative controls were evaluated for vascular effects. The IS scores for the positive controls, NaOH 0.1 M *w*/*v* and sodium lauryl sulfate 1% *w*/*v*, were 13.26 and 10.51, respectively, meaning that these can be considered as extreme irritants (Table 3). Additionally, as expected, the negative controls, NaCl 0.9% *w*/*v* and olive oil, presented no irritant reactions. Furthermore, for NE_B_, no irritant effect was evidenced, suggesting that the surfactants are safe for topical application (Figure 5). Regarding ASE, no vascular reaction was noted, whether or not it was associated with a nanostructure (NE_ASE_) (Table 3 and Figure 5). These results are promising as the nanoemulsion containing ASE showed an absence of vascular alterations in the CAM, which are the first changes associated with skin irritation, in comparison with the negative control NaCl 0.9% *w*/*v* (*p* ≥ 0.05) (Table 3 and Figure 5). The non-irritative potential shown above, added to the results obtained with cell viability assessments, suggests that the developed ASE-loaded nanoemulsion can be safely applied by the topical route [35], including application to damaged skin.

## 4. Conclusions

An ASE-loaded nanoemulsion was prepared in order to investigate the in vitro cytotoxicity profile, proliferative effect, and migration ability in immortalized human keratinocytes (HaCaT cell line), as well as the HET-CAM irritant potential. The results showed an increase in cell viability when ASE was incorporated in nanoemulsions (NE_ASE_). The evaluation of PI incorporation indicated that there was no cell death due to necrosis when treated with NE_ASE_ and suggested that the incorporation of the extract into the nanoemulsion may decrease the ASE toxicity induced by necrosis and increase its proliferation ability. Additionally, the scratch assay indicated that NE_ASE_ was able to increase the cell migration rate and wound closure when compared to ASE and thus may play an important role in topical wound healing activity. We can also verify that the ASE and NE_ASE_ did not present an irritant potential, as demonstrated by the HET-CAM test. In summary, these results indicate the potential of the proposed formulation for topical use in wound healing, in view of the promising effects regarding proliferation and migration in keratinocytes, combined with the suggestive absence of cytotoxicity in vitro and the non-irritant potential.

## Figures and Tables

**Table 1:** Physicochemical characterization of nanoemulsions.

	NE_B_	NE_ASE_
Size (nm)	200 ± 20	307 ± 11.0
Polydispersity index	0.06 ± 0.01	0.12 ± 0.01
ζ-potential (mV)	−21.17 ± 3.21	−43.90 ± 3.92
Viscosity (cP)	1.66 ± 5.09	2.32 ± 2.9
Flavonoids content (μg/mL)	-	1130.3 ± 7.5 *

NE_B_: Blank nanoemulsion; NE_ASE_: nanoemulsion containing *Achyrocline satureioides* extract. * The amount of each isolated flavonoid found was about 315 µg/mL of quercetin, 160 µg/mL of luteolin, and 618 µg/mL of 3-*O*-methylquercetin.

**Table 2:** Morphological alterations and PI incorporation in HaCaT cells (immortalized human keratinocytes) after 24 h of treatment with ASE and NE_ASE_ at 0.625, 1.25, 2.5, 5, and 10 µg/mL concentrations.

Treatments		Concentrations (µg/mL)					
		Control	0.625	1.25	2.5	5	10
ASE	M	No	No	No	Yes	Yes	Yes
	PI	No	No	No	Yes	Yes	Yes
NE_ASE_	M	No	No	No	No	No	No
	PI	No	No	No	No	No	No

Appropriate vehicle controls with DMSO 0.01% (*v*/*v*) and NE_B_ were performed for ASE and NE_ASE_ treatments, respectively. M: morphological alterations (cell elongation and crenation); PI: propidium iodide incorporation; No: absent; Yes: present; ASE: *Achyrocline satureioides* extract; NE_ASE_: *Achyrocline satureioides* extract-loaded nanoemulsion; controls DMSO (0.01%) and NE_B_: blank nanoemulsion.

**Table 3:** Irritant score and irritancy classification in the hen’s egg chorioallantoic membrane test (HET-CAM) (*n* = 5).

Substance	IS (RSD %)	Classification
NaCl 0.9% *w/v*	0 (0)	Non-irritant
NaOH 0.1 M	13.26 (1.41) *	Extreme irritant
Sodium lauryl sulfate 1% *w/v*	10.51 (2.29) *	Extreme irritant
Olive oil	0 (0)	Non-irritant
ASE:Olive oil (1:100) *w/v*	0 (0)	Non-irritant
NE_B_	0 (0)	Non-irritant
NE_ASE_	0 (0)	Non-irritant

IS: irritation score; RSD %: relative standard deviation in percentage; ASE: *Achyrocline satureioides* extract; NE_B_: blank nanoemulsion; NE_ASE_: *Achyrocline satureioides* extract-loaded nanoemulsion. * Statistically different from NaCl 0.9% *w*/*v* (negative control group) (*p* < 0.05).
